# Comparing spousal agreement on perceived responsibility for household natural hazard preparedness to actual behavior

**DOI:** 10.1371/journal.pone.0221217

**Published:** 2019-08-14

**Authors:** Li-San Hung

**Affiliations:** Department of Geography, National Taiwan Normal University, Taipei, Taiwan; Middlesex University, UNITED KINGDOM

## Abstract

This study compares husbands' and wives' views on the person in a couple who should be responsible for preparing for hurricane hazards; it also examines whether the varying levels of agreement reached by husbands and wives regarding this responsibility are associated with actual preparedness behaviors. An online survey targeting married, heterosexual couples living in Sarasota County, Florida, USA was sent out between March and May, 2015. Both the husbands and the wives were asked to fill out the survey. A total of 170 surveys were used for analysis. Results suggested that husbands and wives felt that they had shared responsibility for most of the 19 preparedness behaviors considered. However, a few stereotypically masculine preparedness behaviors were found to typically fall to husbands. Husbands' and wives' views of perceived responsibility were not statistically different, but husbands tended to favor individual responsibility, while wives tended to favor joint responsibility. Higher levels of agreement were significantly associated with greater engagement in planning-related preparedness behaviors. Policy implications are discussed.

## Introduction

Natural disasters cause human fatalities, economic loss, social problems, population displacement, and environmental damage, thus resulting in unsustainable development [1]. Hazard mitigation activities, including preparedness activities, play important roles in sustainable development [2]. Household natural hazard preparedness has been championed by researchers and practitioners as a method of reducing people’s losses from natural disasters. There is established literature on household natural hazard preparedness, but studies thus far have seldom considered household decision-making regarding natural hazard preparedness in either the familial or household context (see exceptions like [3]). In particular, this study addresses: (1) married couples’ views of and levels of agreement about perceived responsibility for preparing for hurricane hazards; and (2) whether these levels of agreement are associated with actual preparedness behaviors. The next section provides a brief literature review of studies on household natural hazard preparedness, as well as couples’ views of and levels of agreement. This section is followed by research methods, results, the discussion, and the conclusion.

## Literature review

### Household natural hazard preparedness

Hazard preparedness encompasses the pre-disaster actions that provide the human and material resources needed to support active responses when disasters occur [4]. Hazard preparedness is aimed at various targets, including businesses [5], communities [6], households/families [7,8], and individuals [9]. At the household level, researchers have been eager to understand whether households are prepared for natural hazards, examining levels of preparedness by asking household members if they have prepared specific items, such as water or canned foods, or have engaged in specific behaviors, such as developing a household evacuation plan [10]. Most of these studies have suggested that households are ill-prepared for natural hazards [10,11].

Aiming to increase the overall level of household natural hazard preparedness, studies have examined the factors that influence preparedness levels. To date, studies have proposed various factors [10,12,13], including cognitive/psychological factors such as fear, risk perception, outcome expectancy, self-efficacy, and protective response costs [14–16]; social factors such as place attachment [17]; political factors, such as trust in public natural hazard protection [18]; and geographical factors, such as hazard exposure [19]. Many of the studies have also examined the relationship between household natural hazard preparedness and household/family characteristics, such as home ownership, number of children, and sociodemographic factors such as age [20] and educational attainment [10]. The results of the examination of the relationships between abovementioned factors and household natural hazard preparedness vary, however [21].

One of the less studied topics in household natural hazard preparedness is the decision-making process that leads to household natural hazard preparedness [12]. To better understand the underlying process that leads to preparedness behaviors, one needs to take into account the interactions and communications among household members, or intra-household dynamics, surrounding household natural hazard preparedness issues [22,23]. For example, Hung [12] divided the household decision-making process regarding household hurricane preparedness into three stages–problem initiation, information search and evaluation, and final decisions–and found that at least half of the married heterosexual couples who were surveyed indicated that they made joint decisions throughout the three stages. Studies have suggested that household natural hazard preparedness behaviors are also related to household members’ gender roles, or divisions of labor [3,23,24]. Finally, some qualitative research on natural hazard preparedness, such as that of Goodman and Cottrell [25], has considered the importance of spousal agreement for household natural hazard preparedness decision-making. To the author’s knowledge, however, there are no studies in either natural hazard or natural disaster research that have systematically examined couples’ views and levels of agreement, or the relationship between levels of agreement and actual behaviors. Questions about the influence of levels of spousal agreement on actual household natural hazard related behaviors, such as evacuation and preparedness, thus remain unanswered.

### Couples’ views and levels of agreement

Gender differences in human behavior have attracted the attention of scholars from various academic disciplines, including sociology, economics, psychology, and behavioral science [26–28]. As heterosexual couples remain the predominant type of couple in many countries, many studies have investigated the differences between husbands and wives, such as married couples’ views of and levels of agreement regarding family- or household-related topics. Researchers in family studies believe that spousal consensus is an important factor for understanding marital relations [29]. As systems theory [30] and family systems theory [31] suggest, spouses influence each other’s attitudes and behaviors. As a result, it is important to consider the views and influence of spouses regarding household-related topics. A couple’s level of agreement or consensus is usually measured by either the events or conditions experienced by both partners, such as household decision-making or conflict resolution, or by the similarities of the partners’ attitudes on topics such as family planning [29]. Couple-level data addresses methodological issues that occur in individual-level data, reveals gender differences on targeted topics [32], and in some cases, shows that spouses’ agreement (or disagreement) is a predictor of household issues.

Some of the studies by family researchers have examined topics including the relationship between marital happiness and spousal consensus on beliefs about marital conflict [33], as well as marital satisfaction and spouses’ attitudinal similarity regarding religious beliefs and marital roles [34]. In fertility studies, understanding spouses’ levels of agreement regarding fertility issues is important because disagreement may hinder fertility decisions, and when disagreement exists, surveying one person on the topic produces only partial information [35,36]. In a study on household decision-making, it was found that about 24 percent of the surveyed couples would be misrepresented in decision-making if only one person within the couple was surveyed due to discrepancies in reporting [37]. In terms of household division of labor, Kamo [38] found that husbands tend to overestimate their own contributions to household work (or wives underestimate the husbands’ contributions) but wives do not, and husbands tend to overestimate (or wives underestimate) wives’ contributions to shopping and paying bills. In a study of patients’ well-being, Cremeans-Smith et al. [39] indicated that when a patient and his or her spouse had similar perceptions (i.e., agreement) of the pain severity of osteoarthritis, the patient indicated better well-being than those patients whose spouses underestimated their pain.

In sum, although various studies have looked into the relationship between gender and natural hazards/natural disasters [40–43], few studies have investigated the views of and agreements between married heterosexual partners in relation to natural hazard preparedness decision-making. Using an online survey, this study asked married couples living in Sarasota County, Florida, USA, a hurricane-prone county [44], to answer questions about household hurricane hazard preparedness and to share their views about the person who should be responsible for preparing for hurricanes. The reported results include husbands’ and wives’ views on their perceived responsibility, as well as their levels of agreement and disagreement when considering the perceived responsibility at the couple level. This study also examines whether spouses’ levels of agreement about responsibility predict actual preparedness behaviors. The research questions include:

1. Do husbands and wives have the same views on who should be responsible for specific household hurricane hazard preparedness behaviors?
2. Is there an association between the level of spousal agreement regarding who should be responsible for specific household hazard preparedness behaviors and the actual preparedness behaviors?

## Methods

This study is based in part on the results from the author’s mixed-methods dissertation [45]. Some of the quantitative results from the dissertation have been published in Hung [12], while some of the qualitative results have been published in Hung [23]. As this study used the same survey dataset as that of Hung [12], only brief descriptions of the methods and sample characteristics are given here. Please refer to Hung [12] for more details. This study was approved by The Office for Research Protection at The Pennsylvania State University (IRB#: STUDY00002066). The associated file that contains survey data can be found at S1 File.

### Survey design

An online survey was designed using the online survey platform Qualtrics. This online survey had three parts. Part I asked a couple to answer questions regarding household hurricane preparedness behaviors as well as household characteristics. Household hurricane preparedness behaviors included 19 items or behaviors [20,46,47] (See S1 Table for list). Participants answered “yes” or “no” for each item, indicating whether the household had prepared the item (such as a three-day supply of water) or had undertaken the behavior (such as making a household evacuation plan) for hurricane-related emergencies. In addition to identifying preparedness behaviors, Part I investigated household-level variables, including length of marriage, household size, residence in a mobile home, home ownership or rental, and the presence of very young children (whether a couple has any infants, toddlers, or other preschool-aged children). Husbands and wives jointly answered the questions in Part I.

Part II and Part III had identical questions. If the husband filled out Part II, then the wife was instructed to fill out Part III, and vice versa. Each part contained an identical series of multiple-choice questions regarding the spouses’ views of who should be responsible for the 19 preparedness behaviors discussed in Part I. A specific question in the survey was: “Please indicate who you think should be primarily responsible for each of the following,” and participants were given the 19 preparedness behaviors and four possible answers: “wife,” “husband,” “both equally responsible,” and “I don’t know.” The categories of “wife,” “husband,” and “joint” are widely used for understanding the roles played by husbands and wives in household decision-making [48–50]. In Part II and Part III, the 19 preparedness behaviors addressed in Part I were linguistically transformed from nouns indicating preparedness items or preparedness behaviors to gerund phrases describing preparedness actions. For example, “a can opener” in Part I became “preparing a can opener” in Part II and Part III; “knowledge of how to turn off the utilities” became “knowing how to turn off the utilities” (see S1 Table for full list of wording changes). In addition to perceived preparedness responsibility questions, information regarding participants’ hurricane risk perceptions and their hurricane experiences was collected. Hurricane risk perceptions were measured using four 5-point Likert scale questions regarding the probability and severity of hurricanes striking Sarasota County (revised from [5]). Hurricane experiences were measured using five yes/no questions regarding participants’ hurricane experiences, including their own experiences, their family members’ experiences, and their relatives’ or friends’ experience (revised from [5,51]).

It should be noted that the research design of this study has two limitations: (1) it is impossible to detect whether the questionnaires were actually filled out as instructed, with husbands and wives jointly finishing Part I of the survey and each of the spouses filling out either Part II or Part III independently; (2) the survey procedure that asked husbands and wives to answer jointly in Part I, followed by individually in Part II and Part III, may have led more couples to select “equally responsible.”

### Survey implementation

This online survey was distributed via two methods between March and May 2015. With the help of the Sarasota County Office of Emergency Management and participants’ homeowner associations (HOAs) in Sarasota County, the survey was sent to contact persons in the HOAs and then forwarded to potential participants in each of the HOAs. This first approach, however, generated an unsatisfactorily low response, and Research Now, an online panel company, was employed to obtain a larger sample of Sarasota County married-couple households.

### Analytical methods

All of the analysis was conducted in IBM SPSS Statistics Subscription version 1.0.0.800. In answering the first research question, counts and percentages were used to indicate views regarding responsibility for household hurricane preparedness at both the individual and couple levels. Kappa statistics were used to assess the level of agreement between husbands and wives regarding responsibility for household natural hazard preparedness behaviors. Kappa coefficients were used to adjust the percentages of agreement by taking into account instances of agreement that had happened by chance. The coefficients ranged from 0, indicating complete disagreement, to 1, suggesting perfect agreement. In addition, to understand whether husbands and wives held the same views on perceived responsibility, a chi-square test (2 × 4) for each of the preparedness items/behaviors was performed between spousal views (husbands’ views and wives’ views) and perceived responsibility (perceived wife responsibility, perceived husband responsibility, perceived both responsibility, and uncertain). Statically significant results suggested that husbands’ reviews and wives’ reviews regarding the perceived responsibility for household hurricane preparedness are significantly differ from each other.

To answer the second research question, chi-square tests (2 x 2) were used to examine the association between the level of agreement regarding preparedness responsibility (i.e., agreement and disagreement) and whether the preparedness items were actually secured or preparedness behaviors were actually completed (i.e., prepared or not prepared). The designation of *spousal agreement* on perceived preparedness responsibility was used to indicate that the husband and wife in a given couple both chose “wife,” “husband,” or “both equally responsible” for a given preparedness behavior. *No agreement* indicates that the husband and wife did not choose the same answers, or they both chose “I don’t know.” While both the husband and wife in a given couple choosing “I don’t know” indicates agreement, this agreement was not likely to be associated with actual behaviors. As a result, those cases in which the husband and wife in a given couple both chose “I don’t know” were labeled as indicative of no agreement. To confirm the relationships, logistic regressions were used. These regressions examined whether agreement remained a statistically significant predictor of those preparedness behaviors that had shown statistically significant associations in chi-square tests when controlling for particular variables (see the discussion in the next paragraph). A model comparison approach was used: In Model 1, only the control variables were included as predictors. In Model 2 (block 2), variable agreement was added. Statistically significant results for the difference in chi-squares between Model 1 and Model 2 suggested that incorporating variable agreement led to statistically significant improvement of the model.

Control variables for the logistic regressions included hurricane risk perceptions, hurricane experiences, household size, length of marriage, home ownership or rental, residence in a mobile home, and the presence of very young children. The selection of control variables was based on previous studies investigating household natural hazard preparedness [11,12,16,20,52]. While the latter five control variables are self-explanatory household-level sociodemographic variables, the treatment of hurricane risk perceptions and hurricane experiences, which were individual-level variables originally, needs more explanation. In accordance with the existing literature [53,54], the following strategy for creating couple-level variables based on individual-level data was used: For both hurricane risk perceptions and hurricane experiences, summative indices from husbands’ answers and wives’ answers were created. Then, the average number of hurricane risk perceptions and hurricane experiences between a particular husband and wife was calculated and used as the surrogate index representing hurricane risk perceptions and hurricane experiences for the given household.

## Results

### Descriptive statistics

A total of 305 survey responses were collected. Many of the responses, however, were not included in the analysis because the responses were either incomplete or identified as likely to have been filled out by only one participant in the household. Overall, 21 responses from HOAs and 149 responses from Research Now were available for analysis. As independent t-tests showed that the two samples did not statistically differ in terms of husbands’ age, wives’ age, husbands’ educational attainment, and wives’ educational attainment (data not shown), the two samples were than combined into one sample for further analysis (N = 170 couples).

Most of the surveyed households (84%) consisted of only two people–the surveyed married couple. About 13% of the households had children under 18 years old, and about 5% of the households included infants, toddlers, or preschool-aged children. Almost all of the surveyed married couples (95%) owned the houses they lived in, and only 8% of the surveyed married couples lived in mobile homes. For length of marriage, about 28% of the survey couples had been married 20 years or fewer, about 31% of the couples had been married for 21 to 40 years, and about 42% of the couples had been married for 41 to 60 years. More than half (60%) of the surveyed husbands were 66 years old or older, and many of them (57%) had at least a bachelor’s degree. Almost all of the husbands were non-Hispanic white. And about 70% of them were retired. For the surveyed wives, nearly half (44%) of them were 66 old or older, with similar percentage of them (43%) had at least a bachelor’s degree. The surveyed wives were also predominately non-Hispanic white. And about half of them (52%) were retired. Our sample is generally reflective of the demographics of Sarasota County. However, the dataset does have some differences when compared to the 2010 census data: This dataset contained fewer married-couple households with children, had significantly more male participants who are 66 years old or older, had fewer female participants between 26–45 years old, and had more female participants between 45–65 years old. For detailed information regarding the descriptive analysis of the sociodemographic variables and the comparison between the sample dataset and 2010 U.S. Census, see Hung [12].

### Hurricane risk perceptions and hurricane experiences

Husbands (M = 12.59, SD = 2.64) and wives (M = 12.56, SD = 2.48) in this study had very similar levels of hurricane risk perceptions. The average number of hurricane risk perceptions between a particular husband and wife was calculated and used as the surrogate index representing the hurricane risk perceptions for the given household (M = 12.57, SD = 2.34). Husbands (M = 1.22, SD = 1.04) and wives (M = 1.25, SD = 1.06) also had very similar levels of hurricane experiences. The average number of hurricane experiences between a particular husband and wife was calculated and used as the surrogate index representing the hurricane experiences for the given household (M = 1.24, SD = 1).

### Preparedness behaviors

Households in this sample had, on average, secured or completed 12.61 preparedness items/behaviors from the list of 19 preparedness items/behaviors. Table 1 shows the percentage of households that had prepared each of the specific items/behaviors. The most common preparedness items/behaviors were a can opener (98%), knowledge of how to turn off utilities (91%), and a three-day supply of medicine (91%). The least common preparedness items/behaviors were an electric generator (24%), a whistle and/or distress flag (24%), and a roof anchor (26%). The above three preparedness items/behaviors had lowest preparedness rates, likely due to the fact that they are hurricane-specific preparedness, compared to other items that are more likely to be commonly seen household items [12].

**Table 1:** Counts and percentages of households that had prepared each household hurricane preparedness item/behavior.

No.	Item/behavior	Counts of households that had prepared it	Percentage of households that had prepared it	No.	Item/behavior	Counts of households that had prepared it	Percentage of households that had prepared it
1	Water	127	74.7	11	Whistle	41	24.1
2	Canned food	140	82.4	12	Charcoal	87	51.2
3	Medicines	154	90.6	13	Evacuation plan	86	50.6
4	Can opener	166	97.6	14	Emergency contact	126	74.1
5	Rainwear	139	81.8	15	Roof anchor	44	25.9
6	Sleeping bags	123	72.4	16	Insurance	123	72.4
7	Utilities on/off	155	91.2	17	Evacuation zone	117	68.8
8	Shutters	104	61.2	18	Yard clearing	125	73.5
9	Electric generator	40	23.5	19	Gas	126	74.1
10	Fire extinguisher	120	70.6				

Note: The names of preparedness items and behaviors are abbreviated. The full names can be found in the S1 Table by referencing each item/behavior’s number.

### Couples’ views of perceived responsibility for household hurricane preparedness behaviors

Table 2 shows the surveyed husbands’ and wives’ views, in counts and percentages, of who should be responsible for the 19 household preparedness items/behaviors. The results shown in Table 2 indicate that for the 19 preparedness items/behaviors considered in this survey, both the husbands and wives surveyed thought that both members of the couple should be held equally responsible for most of the behaviors: over 50% of both husbands and wives chose the “both equally responsible” answer for 15 out of the 19 preparedness behaviors. Evacuation-related behaviors, such as “making a family evacuation plan,” “having an emergency contact outside of the family,” and “knowing the evacuation zone for your family,” garnered responses greater than 80% for “both equally responsible,” making them the highest percentages of all “both equally responsible” answers. Many of the survival items, such as “preparing a three-day supply of water” and “preparing sleeping bags or extra bedding,” had more than 70% “both equally responsible” answers. In contrast, tool-oriented and heavy items, such as “preparing an electric generator,” “preparing shutters for windows or stormproof windows,” and “preparing a roof anchor,” had lower percentages of “both equally responsible” answers and higher percentages of “husband-responsible” answers.

**Table 2:** Couples’ views on perceived responsibility for household hurricane preparedness behaviors.

	Item/Behavior	Perceived Wife responsibility					Perceived Husband Responsibility					Both Equally Responsible					I Don’t Know				
		H		W		Total	H		W		Total	H		W		Total	H		W		Total
		n	%	n	%	%	n	%	n	%	%	n	%	n	%	%	n	%	n	%	%
1	Water	26	15	22	13	14	16	9	11	6	8	126	74	135	79	77	2	1	2	1	1
2	Canned food	40	24	35	21	22	8	5	6	4	4	121	71	127	75	73	1	1	2	1	1
3	Medicines	27	16	26	15	16	8	5	7	4	4	134	79	135	79	79	1	1	2	1	1
4	Can opener	43	25	30	18	21	8	5	9	5	5	117	69	129	76	72	2	1	2	1	1
5	Rainwear	34	20	22	13	16	10	6	10	6	6	124	73	135	79	76	2	1	3	2	1
6	Sleeping bags	36	21	26	15	18	11	6	11	6	6	120	71	131	77	74	3	2	2	1	1
7	Utilities on/off	3	2	1	1	1	63	37	58	34	36	102	60	108	64	62	2	1	3	2	1
8	Shutters	1	1	1	1	1	89	52	81	48	50	76	45	84	49	47	4	2	4	2	2
9	Electric generator	0	0	0	0	0	91	54	81	48	51	63	37	71	42	40	15	9	17	10	9
10	Fire extinguisher	7	4	4	2	3	47	28	36	21	24	113	66	125	74	70	3	2	5	3	2
11	Whistle	16	9	14	8	9	22	13	20	12	12	117	69	122	72	71	14	8	13	8	8
12	Charcoal	2	1	1	1	1	78	46	61	36	41	84	49	103	61	55	6	4	5	3	3
13	Evacuation plan	8	5	7	4	4	19	11	14	8	10	141	83	146	86	84	2	1	3	2	1
14	Emergency contact	21	12	15	9	11	3	2	4	2	2	143	84	148	87	86	3	2	3	2	2
15	Roof anchor	0	0	1	1	0	83	49	76	45	47	59	35	65	38	36	28	16	28	16	16
16	Insurance	12	7	10	6	6	31	18	29	17	18	124	73	126	74	74	3	2	5	3	2
17	Evacuation zone	5	3	2	1	2	14	8	10	6	7	149	88	155	91	89	2	1	3	2	1
18	Yard clearing	1	1	2	1	1	51	30	43	25	28	117	69	123	72	71	1	1	2	1	1
19	Gas	5	3	3	2	2	49	29	37	22	25%	115	68	128	75	71	1	1	2	1	1

Notes: H = husbands’ responses; W = wives’ responses; The names of preparedness items and behaviors are abbreviated. The full names of the items and behaviors can be found in the S1 Table by referencing each item/behavior’s number; Husbands, n = 170, and wives, n = 170, except for no. 9 and no. 11, where husbands, n = 169, and wives = 169, total husbands and wives, n = 340, except for no. 9 and no. 11, where total husbands and wives, n = 338.

At the aggregate level, the highest percentage in the “wife responsible” category was “preparing a three-day supply of canned food” (22%), and the lowest was “preparing an electric generator” (0%). The highest percentage in the “husband responsible” category was “preparing an electric generator” (51%), and the lowest was “having an emergency contact outside of the family” (2%). The highest percentage in the “both equally responsible category” was “knowing the evacuation zone for your family” (89%), and the lowest percentage was “preparing a roof anchor” (36%).

Table 3 shows the discrepancies (subtracting wives’ responses from husbands’ responses) in the couples’ views and in the chi-square test results between husbands’ views and wives’ views. Since the numbers come from subtracting wives’ responses from husbands’ responses, positive discrepancies in the “wife” category indicate that more husbands than wives think that wives are responsible for these behaviors, and negative discrepancies mean that more wives than husbands think that wives are responsible for these behaviors. Positive discrepancies in the “husband” category mean that more husbands than wives believe it is the husbands’ responsibility for these preparedness behaviors, and negative discrepancies mean that more wives than husbands believe it is husbands’ responsibility for these preparedness behaviors. Positive discrepancies in the “both equally responsible” category suggest that more husbands than wives believe the responsibility to prepare falls equally upon spouses, while negative discrepancies suggest that more wives than husbands believe this. None of the nineteen chi-square tests showed significant results, suggesting that husbands’ views and wives’ views regarding the perceived responsibility for household hurricane preparedness do not significantly differ from each other. However, there were discrepancies in the couples’ views that seem to suggest a trend. On the one hand, more wives than husbands think that both spouses are responsible for hurricane preparedness behaviors, even in the case of those items/behaviors considered to be stereotypically masculine, such as “preparing an electric generator” or “preparing a roof anchor.” This point is supported by the fact that negative discrepancies were found in all 19 preparedness behaviors for the “both equally responsible” category. The largest discrepancies were seen in “preparing charcoal, lighter, and a grill,” for which 49% of husbands and 61% of wives suggested that spouses had equal responsibility to prepare (percentages given in Table 2). On the other hand, when preparedness responsibilities were not considered as joint responsibilities but rather as individual responsibilities, more husbands than wives considered it their responsibility or their wives’ responsibility to prepare. This point is supported by the fact that 15 out of the 19 preparedness behaviors showed positive discrepancies in both the “husband responsible” category and the “wife responsible” category. Moreover, the largest discrepancy was found to be in “preparing a charcoal, lighter, and a grill,” which 46% of the husbands, but only 36% of the wives, believed it was their responsibility to prepare (percentages given in Table 2). Specifically, it is noteworthy that more husbands thought it was their responsibility to engage in stereotypically masculine preparedness behaviors, such as “preparing an electric generator” or “acquiring a fire extinguisher.” At the same time, more husbands thought it was their wives’ responsibility to undertake traditionally feminine preparedness behaviors, including many of those related to survival items, like “preparing a can opener” or “preparing rainwear or other protective clothing.” Note that more husbands than wives also thought that it was their wives’ responsibility to establish an emergency contact outside of a family.

**Table 3:** Discrepancies in husbands’ views and wives’ views of perceived responsibility for preparing for hurricane hazards and chi-square test results between husbands’ views and wives’ views.

	Items/Behaviors	Husbands’ View–Wives’ View				*X*^*2*^ results between husbands’ views and wives’ views
		Wife	Husband	Both Equally Responsible	I Don’t Know	
1	Water	4	5	−9	0	*X*^*2*^ (3, *n* = 340) = 1.57, *p* = 0.666
2	Canned food	5	2	−6	−1	*X*^*2*^ (3, *n* = 340) = 1.098, *p* = 0.778
3	Medicines	1	1	−1	−1	*X*^*2*^ (3, *n* = 340) = 0.423, *p* = 0.936
4	Can opener	13	−1	−12	0	*X*^*2*^ (3, *n* = 340) = 2.959, *p* = 0.398
5	Rainwear	12	0	−11	−1	*X*^*2*^ (3, *n* = 340) = 3.239, *p* = 0.356
6	Sleeping bags	10	0	−11	−1	*X*^*2*^ (3, *n* = 340) = 2.295, *p* = 0.513
7	Utilities on/off	2	5	−6	−1	*X*^*2*^ (3, *n* = 340) = 1.578, *p* = 0.664
8	Shutters	0	8	−8	0	*X*^*2*^ (3, *n* = 340) = 0.776, *p* = 0.855
9	Electric generator	0	10	−8	−2	*X*^*2*^ (3, *n* = 338) = 1.184, *p* = 0.553
10	Fire extinguisher	3	11	−12	−2	*X*^*2*^ (3, *n* = 340) = 3.381, *p* = 0.337
11	Whistle	2	2	−5	1	*X*^*2*^ (3, *n* = 338) = 0.37, *p* = 0.946
12	Charcoal	1	17	−19	1	*X*^*2*^ (3, *n* = 340) = 4.434, *p* = 0.218
13	Evacuation plan	1	5	−5	−1	*X*^*2*^ (3, *n* = 340) = 1.111, *p* = 0.774
14	Emergency contact	6	−1	−5	0	*X*^*2*^ (3, *n* = 340) = 1.229, *p* = 0.746
15	Roof anchor	−1	7	−6	0	*X*^*2*^ (3, *n* = 340) = 1.598, *p* = 0.66
16	Insurance	2	2	−2	−2	*X*^*2*^ (3, *n* = 340) = 0.764, *p* = 0.858
17	Evacuation zone	3	4	−6	−1	*X*^*2*^ (3, *n* = 340) = 2.271, *p* = 0.518
18	Yard clearing	−1	8	−6	−1	*X*^*2*^ (3, *n* = 340) = 1.498, *p* = 0.683
19	Gas	2	12	−13	−1	*X*^*2*^ (3, *n* = 340) = 3.203, *p* = 0.361

Note: Preparedness items and behaviors are abbreviated. Check the full names of the items and behaviors in the S1 Table by referencing the NO.

### Spousal agreement (or disagreement) about responsibility for household hurricane preparedness behaviors

Table 4 shows the level of agreement (or disagreement) on household hurricane-preparedness responsibility between a husband and a wife in a given family. The results show that for each of the 19 preparedness behaviors, at least 56% of the husbands and wives agreed on who was primarily responsible. The level of agreement ranged from 56% (“preparing a roof anchor”) to 89% (“knowing the evacuation zone for your family”). In addition, for 16 out of the 19 preparedness behaviors, the majority of couples agreed that the husband and wife are equally responsible for the given behavior. For the remaining three preparedness behaviors–“preparing shutters for windows or stormproof windows” (37%), “preparing an electric generator” (38%), and “preparing a roof anchor” (31%)–the majority of husbands and wives agreed that the husbands are primarily responsible. There was no specific item for which both husbands and wives thought that the wives are primarily responsible. The highest percentage of agreement among those behaviors deemed “wife responsible” preparedness behaviors was found for “preparing a three-day supply of canned food” (15%). It is worth noting that the three “husband responsible” items about which couples agreed showed a high percentage of “no agreement.”

**Table 4:** Levels of agreement on perceived responsibility for preparedness behaviors and Kappa coefficients.

	Items/Behaviors	Agreement (%)				No Agreement (%)	D (%)	Kappa
		W	H	B	Total			
1	Water	7.6	4.1	68.2	80.0	19.4	0.6	0.496
2	Canned food	14.7	2.4	64.7	81.8	17.6	0.6	0.578
3	Medicines	8.8	1.2	71.2	81.2	18.2	0.6	0.476
4	Can opener	11.8	2.4	61.8	75.9	23.5	0.6	0.453
5	Rainwear	8.2	2.9	65.3	76.5	22.9	0.6	0.414
6	Sleeping bags	9.4	3.5	62.9	75.9	23.5	0.6	0.433
7	Utilities on/off	0.6	21.8	47.6	70.0	28.8	1.2	0.414
8	Shutters	0.6	36.5	32.9	70.0	28.8	1.2	0.455
9	Electric generator	0	36.7	27.8	64.5	30.2	5.3	0.476
10	Fire extinguisher	1.2	13.5	57.6	72.4	26.5	1.5	0.413
11	Whistle	5.3	5.9	60.9	72.2	24.9	3	0.472
12	Charcoal	0.6	29.4	42.9	72.9	25.3	1.8	0.527
13	Evacuation plan	1.8	4.1	77.6	83.5	15.9	0.6	0.436
14	Emergency contact	5.9	1.2	80.6	87.6	11.2	1.2	0.564
15	Roof anchor	0	31.2	24.7	55.9	33.5	10.6	0.461
16	Insurance	3.5	10	64.7	78.2	20	1.8	0.528
17	Evacuation zone	1.2	2.9	84.7	88.8	10	1.2	0.488
18	Yard clearing	0.6	17.6	60	78.2	21.2	0.6	0.503
19	Gas	1.8	15.9	61.8	79.4	20	0.6	0.532

Note: W means that the husband and wife in a given couple both chose “wife”; H means that the husband and wife in a given couple both chose “husband”; B means that the husband and wife in a given couple both chose “both equally responsible”; D means that that the husband and wife in a given couple both chose “I don’t know.” All the Kappa coefficients are significant (*p* < .001); Preparedness items and behaviors are abbreviated. Check the full names of the items and behaviors in the S1 Table by referencing the NO.

Kappa coefficients for the items are listed in Table 3. Because of the nature about how Kappa coefficient is calculated, the situation when both husband and wife in a given couple chose “I don’t know” was treated as an agreement when calculating Kappa coefficients. The Kappa coefficients ranged from 0.413 (“fire extinguisher”) to 0.578 (“preparing a three-day supply of canned food”). Some of the items that had higher Kappa coefficients include: “preparing a three-day supply of canned food” (0.578); “having an emergency contact outside of the family” (0.564); “filling the car’s gas tank” (0.532); “purchasing flood and/or wind insurance” (0.528); “preparing charcoal, lighter, and a grill” (0.527); and “clearing your yard of potential airborne items” (0.503). On the other hand, some of the items that had lower Kappa coefficients include “having a fire extinguisher” (0.413); “preparing rainwear or other protective clothing” (0.414); “knowing how to turn off the utilities” (0.414); “preparing sleeping bags or extra bedding” (0.433); and “making a family evacuation plan” (0.436). Overall, the Kappa coefficients indicated moderate agreement between husbands and wives about who should be responsible for preparing specific items.

### Couples’ level of agreement and actual preparedness behaviors

Nineteen (19) chi-square tests were conducted, one for each of the preparedness items, to examine the association between level of agreement about perceived preparedness responsibility and whether the preparedness item was actually secured or the preparedness behavior was actually undertaken. The results are presented in Table 5.

**Table 5:** Chi-Square results for association between level of agreement and actual behaviors.

NO	Items/Behaviors		No Agreement	Agreement	*X*^*2*^ Tests
1	Water	Not Prepared	10 (29.4)	33 (24.3)	*X*^*2*^ (1, *n* = 170) = 0.381, *p* = .537
		Prepared	24 (70.6)	103 (75.7)	
2	Canned Food	Not Prepared	5 (16.1)	25 (18)	*X*^*2*^ (1, *n* = 170) = 0.06, *p* = .806
		Prepared	26 (83.9)	114 (82)	
3	Medicines	Not Prepared	4 (12.5)	12 (8.7)	*X*^*2*^ (1, *n* = 170) = 0.441, *p* = .507
		Prepared	28 (87.5)	126 (91.3)	
4	Can Opener	Not Prepared	0 (0)	4 (3.1)	*X*^*2*^ (1, *n* = 170) = 1.302, *p* = .254
		Prepared	41 (100)	125 (96.9)	
5	Rainwear	Not Prepared	9 (22.5)	22 (16.9)	*X*^*2*^ (1, *n* = 170) = 0.638, *p* = .424
		Prepared	31 (77.5)	108 (83.1)	
6	Sleeping Bags	Not Prepared	10 (24.4)	37 (28.7)	*X*^*2*^ (1, *n* = 170) = 0.286, *p* = .592
		Prepared	31 (75.6)	92 (71.3)	
7	Utilities On/Off	Not Prepared	7 (13.7)	8 (6.7)	*X*^*2*^ (1, *n* = 170) = 2.176, *p* = .14
		Prepared	44 (86.3)	111 (93.3)	
8	Shutters	Not Prepared	22 (43.1)	44 (37)	*X*^*2*^ (1, *n* = 170) = 0.571, *p* = .45
		Prepared	29 (56.9)	75 (63)	
9	Electric Generator	Not Prepared	49 (80.3)	81 (74.3)	*X*^*2*^ (1, *n* = 169) = 0.787, *p* = .375
		Prepared	12 (19.7)	27 (25.7)	
10	Fire Extinguisher	Not Prepared	15 (31.9)	35 (28.5)	*X*^*2*^ (1, *n* = 170) = 0.196, *p* = .658
		Prepared	32 (68.1)	88 (71.5)	
11	Whistle	Not Prepared	39 (81.3)	90 (73.8)	*X*^*2*^ (1, *n* = 169) = 1.053, *p* = .305
		Prepared	9 (18.8)	32 (26.2)	
12	Charcoal	Not Prepared	24 (52.2)	59 (47.6)	*X*^*2*^ (1, *n* = 170) = 0.283, *p* = .595
		Prepared	22 (47.8)	65 (52.4)	
13	Evacuation Plan	Not Prepared	17 (60.7)	67 (47.2)	*X*^*2*^ (1, *n* = 170) = 1.713, *p* = .191
		Prepared	11 (39.3)	75 (52.8)	
14	Emergency Contact	Not Prepared	10 (47.6)	34 (22.8)	*X*^*2*^ (1, *n* = 170) = 5.901, *p* = .015
		Prepared	11 (52.4)	115 (77.2)	
15	Roof Anchor	Not Prepared	56 (74.7)	70 (73.7)	*X*^*2*^ (1, *n* = 170) = 0.021, *p* = .885
		Prepared	19 (25.3)	25 (26.3)	
16	Insurance	Not Prepared	16 (43.2)	31 (23.3)	*X*^*2*^ (1, *n* = 170) = 5.751, *p* = .016
		Prepared	21 (56.8)	102 (76.7)	
17	Evacuation Zone	Not Prepared	10 (52.6)	43 (28.5)	*X*^*2*^ (1, *n* = 170) = 4.589, *p* = .032
		Prepared	9 (47.4)	108 (71.5)	
18	Yard Clearing	Not Prepared	7 (18.9)	38 (28.6)	*X*^*2*^ (1, *n* = 170) = 1.386 *p* = .239
		Prepared	30 (81.1)	95 (71.4)	
19	Gas	Not Prepared	8 (22.9)	36 (26.7)	*X*^*2*^ (1, *n* = 170) = 0.21, *p* = .647
		Prepared	27 (77.1)	99 (73.3)	

Note: Numbers in parentheses in the “no agreement” and “agreement” columns represent the percentage for each cell relative to the other cells in the same column.

Among the tests, 3 out of the 19 preparedness items, all of which were planning-related preparedness behaviors, showed statistical significance: “having an emergency contact outside of the family,” “purchasing flood and/or wind insurance,” and “knowing the evacuation zone for your family.” A cross-tabulation indicated that for all three of these preparedness items, higher percentages of agreement were related to higher percentages of actual preparedness behaviors. For example, among the couples who agreed on who should be responsible for establishing an emergency contact, 77% of them said that they actually had emergency contacts outside of their families. On the other hand, for those couples who did not agree, only 52% said that they had emergency contacts.

To verify the relationships, six (6) logistic regressions were performed, using the preparedness behaviors “having an emergency contact outside of the family,” “purchasing flood and/or wind insurance,” and “knowing the evacuation zone for your family” as dependent variables. In predicting each of the three aforementioned preparedness behaviors, two different regression analyses were performed. In Model 1, only the control variables were included as predictors. In Model 2, variable agreement was included in the analysis.

The results (Table 6) indicate that for each of the three analyses, after controlling for related variables, variable agreement remained a positive and significant predictor of the preparedness behaviors examined. More importantly, incorporating variable agreement statistically improved the model fit for all three analyses.

**Table 6:** Logistic regression results in predicting specific preparedness behaviors.

Analysis		Analysis 1				Analysis 2				Analysis 3			
Dependent variables		Emergency Contact				Insurance				Evacuation Zone			
Models		Model 1		Model 2		Model 1		Model 2		Model 1		Model 2	
**Independent variables**		b	p	b	p	b	p	b	p	b	p	b	p
	**Hurricane risk perception**	.041	.588	.032	.684	.027	.728	.033	.678	.010	.895	.011	.886
	**Hurricane experience**	.318	.100	.305	.124	.138	.472	.117	.554	.298	.107	.275	.144
	**Length of marriage**	.028	.029	.030	.020	.027	.035	.030	.023	.013	.287	.017	.166
	**Household size**	-.143	.670	-.114	.743	-.508	.114	-.440	.179	-.559	.082	-.556	.083
	**Presence of very young children (yes = 1)**	.270	.776	.609	.542	1.138	.247	1.209	.215	-.105	.912	-.017	.986
	**Own/rent home (own = 1)**	-1.375	.218	-1.466	.204	-1.755	.044	1.669	.062	1.000	.215	.947	.249
	**Mobile home (yes = 1)**	-.237	.714	-.332	.613	-1.189	.049	-1.213	.048	-.059	.926	-.040	.951
	**Agreement (yes = 1)**			1.177	.021			.916	.032			1.172	.025
	**Constant**	-.524	.724	-1.271	.418	.372	.803	.033	.752	.520	.719	-.532	.732
**Model statistics** **(CI = conditional index)**		*R*^2^ = .059 (Cox & Snell), .087 (Nagelkerke). Model *X*^2^(7) = 10.382, p = .168. CI = 25.		*R*^2^ = .087 (Cox & Snell), .128 (Nagelkerke). Model *X*^2^(8) = 15.547, p = .049. Change in Chi-square: *X*^2^(1) = 5.165, p = .023. CI = 29.15.		*R*^2^ = .11 (Cox & Snell), .159 (Nagelkerke). Model *X*^2^(7) = 19.827, p = .006. CI = 25.		*R*^2^ = .133 (Cox & Snell), .193 (Nagelkerke). Model *X*^2^(8) = 24.345, p = .002. Change in Chi-square: *X*^2^(1) = 4.518, p = .034. CI = 27.53.		*R*^2^ = .078 (Cox & Snell), .11 (Nagelkerke). Model *X*^2^(7) = 13.88, p = . 053. CI = 25.		*R*^2^ = .105 (Cox & Snell), .148 (Nagelkerke). Model *X*^2^(8) = 18.875, p = .016. Change in Chi-square: *X*^2^(1) = 4.995, p = .025. CI = 28.33	

It is worth noting that the results in Table 5 indicate that agreement was associated with higher percentages of actual completion for 14 of the 19 preparedness behaviors. The five preparedness behaviors that showed counter-effects were “preparing a three-day supply of canned food,” “preparing a can opener,” “preparing sleeping bags or other protective clothing,” “clearing your yard of potential airborne items,” and “filling the car’s gas tank.

## Discussion

The analysis suggests that husbands and wives have moderate levels of agreement on who is responsible for preparing for hurricanes, and husbands’ and wives’ views on who is responsible do not statistically differ from each other. In terms of “having an emergency contact outside of the family,” “purchasing flood and/or wind insurance,” and “knowing the evacuation zone for your family,” which are usually considered planning-related preparedness behaviors [55,56], the results show that higher levels of agreement are associated with higher levels of task and behavior completion.

Both husbands and wives in this study indicated that they felt a shared responsibility for most of the preparedness behaviors, except for those preparedness behaviors that can be characterized as stereotypically masculine. Since couples consider preparing for natural hazards a matter of shared responsibility, couples are very likely to discuss and negotiate with one another about preparedness decisions (as discussed in the household decision-making literatures such as Ashraf [57]). Attention should be paid to better understanding these dynamics. Past research on household behaviors related to natural hazards or natural disasters, such as preparedness or evacuation, has usually assumed that the person who fills out the survey sufficiently represents all of the members of his or her household [16]. More studies considering household natural hazard preparedness as a household or family process are thus needed [3]. An increased focus on understanding the influence of these intra-household dynamics on household behaviors related to natural hazards or natural disasters is also needed [12,23,24]. In this study, several of the planning-related preparedness behaviors, including evacuation preparedness behaviors, had very high percentages of “both equally responsible” answers, suggesting that the execution of these behaviors is especially dependent upon interactions and communications between spouses [24,58]. Stereotypically masculine preparedness behaviors had high percentages of “husband responsible” answers, which is logical due to the stereotypically masculine physical and mechanical dimensions of the behaviors.

The discrepancies between husbands’ views and wives’ views, reported in Table 3, are particularly noteworthy. While the statistical results suggest that husbands’ and wives’ views regarding preparedness behaviors did not differ from each other, the general trend seen in this study is that the wives surveyed tended to favor shared responsibility, while the husbands tended to favor individual responsibility, either theirs or their wives’. This egalitarian perspective might present a problem for preparedness behaviors. That is, the “both responsible” view might lead to situations in which one spouse believes that if he or she does not prepare, his or her partner will, or one spouse thinks that he or she cannot undertake any preparedness action without consulting with his or her partner first [24]. Both thoughts might make people act more passively when approaching preparedness behaviors than they otherwise would, thereby resulting in delayed or unaccomplished actions. Views on the need for interaction before making preparedness decisions are likely to be an especially important issue in emergency situations, during which the time to react is short [24]. The results of this study suggest that more wives than husbands may find themselves in such an emergency situation, but it is worth noting that fairly large percentages of husbands and wives indicated that they believe they share responsibility for preparedness behaviors. The individual view of responsibility suggests that preparedness behaviors should be undertaken independently. Note the tendency for husbands to favor individual responsibility for either themselves or their wives. Situations in which husbands (or wives) consider themselves to have designated preparedness behaviors show consistency, but inconsistency appears when husbands (or wives) consider their spouses to have designated preparedness behaviors. However, the general trend observed in this study is that husbands considered stereotypically masculine preparedness behaviors their responsibility and considered stereotypically feminine preparedness behaviors their wives’ responsibility. While the husbands showed inconsistency in their perceptions of who was responsible for stereotypically feminine preparedness behaviors, this might not be very important because most of the stereotypically feminine preparedness items, such as “preparing a three-day supply of canned food” or “preparing a can opener,” are not preparedness-specific items and are completed in most of the households. Thus, this inconsistency is not likely to result in a lack of preparation.

The association between level of agreement and actual behaviors was significant for certain planning-related preparedness behaviors: “having an emergency contact outside of the family,” “purchasing flood and/or wind insurance,” and “knowing the evacuation zone for the family.” These results again suggest that many planning-related preparedness behaviors are behaviors that are especially tied to interactions among household members, rather than to single individuals’ thinking. However, while the results could be understood as suggesting that agreement in planning-related behaviors leads to actual behaviors, the counterstatement, that the behaviors are completed because agreement is reached, could also be true. Future research is needed to examine the causal effect of agreement on actual preparedness behaviors, especially planning-related behaviors. Note that for 14 of the 19 preparedness behaviors considered in this study, agreement was associated with a higher completion rate; the remaining 5 behaviors that did not show this association were everyday, not preparedness-specific, items, such as “preparing a three-day supply of canned food” or “preparing a can opener.” Negotiations about these items are usually not necessary. In addition, although some studies have suggested that the presence of children, especially very young children, in households is related to lower levels of motivation to prepare, greater perceived difficulty in preparing, greater lack of time to prepare, and even lower preparedness levels than in childless households [59], the results of the logistic regressions shown in Table 6 do not indicate that the presence of infants, toddlers, or preschool-aged children is a statistically significant predictor of the aforementioned planning-related preparedness behaviors. Future studies might examine whether these results are related to the specific preparedness behaviors investigated by this study.

The consideration that spousal agreement may be an important factor affecting household natural hazard preparedness stems from academic fields such as family studies, sociology of the family, and family psychology. The results of this study confirm that couple-level variables are important in preparedness behaviors [3]. Few studies, if any, treat existing individual-level variables that are usually considered important drivers of preparedness behaviors, such self-efficacy, fear, risk perceptions, and experiences, at the couple level in order to understand their influences on household natural hazard preparedness behaviors. In our study, the couple-level hurricane risk perception and hurricane experience variables were not statistically significant predictors of the three preparedness behaviors examined. More studies are needed to establish these variables’ relationships in different contexts.

Practitioners and policymakers should note the different views that husbands and wives might have regarding preparing for natural hazards. High levels of agreement between husbands and wives on the perceived responsibility for preparing for natural hazards are likely to increase levels of household natural hazard preparedness, while disagreements are likely to impede the undertaking of preparedness behaviors (see also Paton [22]). Encouraging couples to discuss preparedness issues, especially planning-related behaviors, is likely to facilitate the completion of preparedness behaviors.

The limitations of using this dataset are discussed in Hung [12]. One of these limitations is the non-representative nature of the dataset. However, the fact that the sample is made up of wealthy, highly educated, and predominantly white elderly retirees means that this study is especially suitable for understanding preparedness behaviors for this demographic group, whether its members reside in Florida or in other Sun Belt states in the southern United States. Also, this study followed some literatures [60–62] and used a dichotomous scale (yes/no) for preparedness behaviors, but the results of the analysis could be differ if a continuous scale (e.g., from never prepare to always prepare) was used for preparedness behaviors, in which other studies have been adopted [63]. In addition, two additional limitations have been discussed in the methods section because of the research design: (1) it is impossible to detect whether the questionnaires were actually filled out as instructed, and (2) answering individual questionnaires after the joint questionnaires may led more couples to select “equally responsible” category when measuring preparedness responsibility.

Future studies can focus on uncovering the factors affecting the agreement between husband and wife on perceived responsibility for preparing for natural hazards. In addition, household decision-making is often influenced by power dynamics or resources controlled by different household members. For instance, the main financial contributor to the family often has greater say in the household decision-making process [64]. Future studies would benefit from addressing how power dynamics influence household decision-making regarding natural hazard preparedness.

## Conclusion

Many of the quantitative, survey-based studies on household natural hazard preparedness take an individual view of factors influencing preparedness behaviors, neglecting the role of couple-level variables in facilitating or impeding preparedness behavior. This study suggests that spousal agreement on perceived responsibility in preparing for natural hazards is a statistically significant predictor of household hurricane preparedness. Given the scarcity of studies that quantitatively examine how couple-level variables influence preparedness behaviors, further research is necessary.

## Supporting information

S1 File Survey data for this study. (SAV)

S1 Table Original and revised questionnaire language. (DOCX)
