# Supplementary material for: Comparing spousal agreement on perceived responsibility for household natural hazard preparedness to actual behavior
Source: PLoS One. 2019 Aug 14;14(8):e0221217. doi: 10.1371/journal.pone.0221217 (PMC6693778; doi:10.1371/journal.pone.0221217)
Supplement: S1 Table — (DOCX) [file pone.0221217.s002.docx]

**S1 Table. Original and revised questionnaire language**

| **NO** | **Preparedness items/behaviors (used in Part I)** | **Wording changes (used in Part II and Part III)** |
| --- | --- | --- |
| **1** | A 3-day supply of water | Preparing a 3-day supply of water |
| **2** | A 3-day supply of canned food | Preparing a 3-day supply of canned food |
| **3** | A 3-day supply of medicines | Preparing a 3-day supply of medicines |
| **4** | A can opener | Preparing a can opener |
| **5** | Rainwear or other protective clothing | Preparing rainwear or other protective clothing |
| **6** | Sleeping bags or extra bedding | Preparing sleeping bags or extra bedding |
| **7** | Knowledge of how to turn off the utilities | Knowing how to turn off the utilities |
| **8** | Shutters for windows or stormproof windows | Preparing shutters for windows or stormproof windows |
| **9** | An electric generator | Preparing an electric generator |
| **10** | A fire extinguisher | Acquiring a fire extinguisher |
| **11** | A whistle and/or distress flag | Preparing a whistle and/or distress flag |
| **12** | Charcoal, lighter, and grill | Preparing charcoal, a lighter, and a grill |
| **13** | A family evacuation plan | Making a family evacuation plan |
| **14** | An emergency contact outside of the family | Having an emergency contact outside of the family |
| **15** | A roof anchor | Anchoring the roof |
| **16** | Flood and/or wind insurance | Purchasing flood and/or wind insurance |
| **17** | Knowledge of the evacuation zone for your family | Knowing the evacuation zone for the family |
| **18** | A yard clear of potential airborne items | Clearing your yard of potential airborne items |
| **19** | A full gas tank in your car | Filling the car’s gas tank |
